# Assessment of Knowledge, Attitude, and Associated Factors of Mpox Among Healthcare Professionals at Debre Tabor Comprehensive Specialized Hospital, Northwest Ethiopia, 2024: A Cross‐Sectional Study

**DOI:** 10.1002/hsr2.70371

**Published:** 2025-01-22

**Authors:** Teklehaimanot Kiros, Mulat Erkihun, Mitikie Wondmagegn, Andargachew Almaw, Ayenew Assefa, Ayenew Berhan, Dessie Tegegne, Alemie Fentie, Tegenaw Tiruneh, Birhanemaskal Malkamu, Mahider Shimelis, Eninur Dejen, Birhanu Getie, Shewaneh Damtie, Yenealem Solomon, Bekele Sharew

**Affiliations:** ^1^ Department of Medical Laboratory Science, College of Health Sciences Debre Tabor University Debre Tabor Ethiopia

**Keywords:** attitude, Ethiopia, healthcare professionals, knowledge, Mpox

## Abstract

**Background:**

Mpox is a zoonotic disease that has become a significant public health concern, especially in regions beyond its usual endemic areas in Africa. The rising global incidence and its classification as a Public Health Emergency of International Concern by the World Health Organization highlight the importance of healthcare professionals (HCPs) being knowledgeable and well‐prepared to effectively manage the virus. This study aims to assess the knowledge, attitudes, and factors associated with HCPs regarding Mpox infections at Debre Tabor Comprehensive Specialized Hospital in Northwest Ethiopia.

**Methods:**

Between June 13th and September 3rd, 2024, a cross‐sectional study was conducted, among 384 HCPs selected through convenience sampling. A pretested, and well‐structured, questionnaire was used to assess the sociodemographic characteristics, knowledge, and attitudes of HCPs. The collected data were analyzed using descriptive statistics and logistic regression. A *p*‐value of ≤ 0.05 was considered a statistically significant association.

**Results:**

Out of the 384 participants, the majority were male (55.47%) and aged 31–39 years (33.85%). Nurses made up the largest professional group at 31.25%. Only 28.13% and 37.76% of HCPs demonstrated good knowledge and favorable attitudes toward the Mpox, respectively. Interestingly, HCPs with prior information sources about Mpox were likely to possess good knowledge (AOR = 1.45, *p* = 0.002).

**Conclusions:**

The findings emphasize important gaps in the knowledge and attitude of HCPs. It is imperative to implement targeted educational initiatives such as information dissemination, training, and continuous professional development to improve the capacity of HCPs to effectively respond to Mpox and other emerging infectious diseases.

**Abbreviations**
DTCSH: Debre Tabor Comprehensive Specialized Hospital
HCWs: healthcare workers
Mpox: Monkeypox virus
PHEIC: public health emergency of international concern
WHO: World Health Organization

## 1 Introduction

The emergence of infectious diseases as a significant public health concern has garnered considerable attention from local and international research and development (R&D) centers. The increase in epidemic and pandemic‐causing microbial pathogens, especially bacterial and viral agents, poses a global public health threat [1, 2]. The evolution of infectious agents, especially viruses, is driven by various factors such as microbial properties (genomic plasticity), host‐pathogen interaction, climate change, socioeconomic conditions, and globalization [3]. Due to existing immunity, the chance of a pandemic caused by viruses already present in humans is low. However, most recorded pandemics and public health emergencies of international concern, including the World Health Organization (WHO) priority diseases, are zoonosis. Smallpox and polio viruses are exceptions, as they only exist in humans, enabling efforts towards their eradication. The number of zoonotic diseases has increased due to factors such as habitat encroachment, wildlife trade, and extensive animal husbandry, leading to more frequent human‐animal contact [4, 5].

To tackle these emerging public health concerns, the WHO has adopted a scientific guideline for pathogen priority during epidemic and pandemic response as well as preparedness. In its 2024 scientific framework for R&D, the Monkeypox (Mpox) virus is considered a public health emergency of concern in terms of its risk priority. The scientific framework presents a thorough strategy aimed at strengthening global health resilience against infectious diseases. It underscores the necessity of collaborative research across various pathogen families, with a focus on priority and prototype pathogens to expedite the development of medical countermeasures, including vaccines, therapeutics, and diagnostics. Key priorities of the framework include enhancing our understanding of pathogen microbiology, pathogenesis, and immunology, as well as developing high‐throughput tools and effective animal models for research purposes. Furthermore, the framework advocates for streamlined clinical trial processes, equitable access to research findings, and the creation of global networks to support data sharing and manufacturing capabilities. Ultimately, the goal is to cultivate adaptable knowledge and resources that can be rapidly mobilized in response to emerging health threats, significantly boosting the world's capacity to detect, prevent, and respond to potential pandemics [6].

The Mpox is a zoonotic disease caused by a virus that has recently gained prominence in global public health. This enveloped double‐stranded DNA virus is classified within the Orthopoxvirus genus of the *Poxvirida*e family [7, 8]. Its oval viral structure features a lipoprotein outer membrane that encases a core containing the genome and essential enzymes for replication. Mpox has two genetic clades: Clade I, which has historically been linked to the Congo Basin (Central Africa) and is known to cause more severe disease and higher mortality rates, and Clade II, which is associated with West Africa and further divided into subclades IIa and IIb. Clade II is generally less severe than Clade I. Recent classifications have also identified that Clade I has been divided into subclades Ia and Ib, contributing to its genetic diversity [9, 10]. The disease has garnered significant attention due to outbreaks, particularly in Central and West Africa, and global cases reported outside these regions in recent years [10]. The symptoms of the Mpox in humans resemble those of smallpox, albeit with a lower mortality rate. Recently, concerns about a potential global pandemic have surged, driven by reports of Mpox cases spreading throughout Africa and beyond. Historically, Mpox was a rare zoonotic disease confined to endemic areas of Western and Central Africa. However, the recent emergence of Mpox cases in diverse regions has heightened worries about its natural evolution. The term “Monkeypox” is thought to originate from the fact that Mpox was first identified in 1958 in laboratory monkeys transported from Singapore [7, 10, 11].

Owing to the increasing number of cases in the African region and beyond, it has been declared a Public Health Emergency of International Concern (PHEIC) by the WHO in its emergency committee meeting held on August 14, 2024 [12]. The WHO's external situation report on the Mpox outbreak, released on August 12, 2024, details several key findings from January 2022 to June 2024. During this period, a total of 99,176 laboratory‐confirmed Mpox cases and 208 fatalities were documented across 116 countries. In June 2024 alone, there were 934 new cases and four deaths, with the majority of cases occurring in the African region (567), followed by the Americas (175) and Europe (100). The report warns that the true number of cases is likely underestimated due to decreasing reporting rates and limited testing, particularly in remote areas of the Democratic Republic of the Congo, which accounted for 96% of the confirmed cases in Africa. Additionally, the report highlights a worrying increase in case counts within the African region, linked to both an expanding outbreak and enhanced access to laboratory confirmation [13].

The disease can transmit and spread from animal to human through direct contact with blood, bodily fluids, or cutaneous or mucosal lesions of infected animals. It is most commonly associated with rodents like rats, mice, and squirrels. Similarly, human to human through close contact with respiratory secretions, skin lesions, or objects contaminated by the virus. Human‐to‐human transmission is less common compared to zoonotic transmission but can occur in household settings, healthcare facilities, or through close, prolonged contact [14, 15]. The signs and symptoms include fever, rash, swollen lymph nodes, and respiratory symptoms. The initial presentations may often begin with fever, headache, muscle aches, and exhaustion. Lymphadenopathy (swollen lymph nodes) is a distinguishing feature of Mpox compared to similar diseases like smallpox. Its incubation period is usually 6–13 days but can range from 5 to 21 days. However, the case fatality rate varies by strain, with the Central African strain being more severe. While most cases are mild, severe cases can occur, particularly in individuals with weakened immune systems or in areas where healthcare access is limited. Complications can include secondary bacterial infections, respiratory distress, and, in rare cases, death [10, 11].

Immune response to Mpox includes both the innate and adaptive immune system. Because of similarities in viruses, people who received smallpox vaccination may have partial cross‐protection against Mpox. Vaccination is also considered key to the prevention of the infection [16, 17]. WHO has recommended primary preventive pre‐exposure vaccination for those whose exposure is high including healthcare professionals and persons having many sexual partners, especially men having sex with men. The second one is the post‐exposure preventive vaccination for contacts of a confirmed case of the virus to prevent or mitigate the illness [18]. Other preventive measures include avoiding close contact with people who have a Mpox‐like rash, fairly frequent handwashing with soap and water, use of personal protective equipment while taking care of individuals infected with the virus, isolating those infected with the intent of containing the virus to prevent its spread, and performing surveillance and contact tracing [19].

To date, no cases of Mpox have been reported in Ethiopia. Published data on the disease, particularly assessment of knowledge, attitude and its determinant factors among healthcare professionals is severely limited with only two studies available [20, 21]. It is crucial to implement public health measures to control the increasing incidence of infectious diseases. For Mpox infection, it is essential to assess the knowledge, attitude, and practices of healthcare professionals due to the rising global prevalence and the significant role they play in managing the pandemic. Previous studies [22, 23, 24, 25] have offered important insights into healthcare professionals' understanding of infections within hospital environments, revealing gaps in knowledge and attitudes that may hinder effective disease management. Furthermore, earlier research may have inadequately considered the swift evolution of disease information, shifts in public health guidelines, or differences in the healthcare workforce's exposure and response strategies. Conducting a targeted study is essential to address these gaps, delivering current, context‐specific data that can guide tailored interventions and improve healthcare professionals' overall readiness to combat Mpox infections [26]. Therefore, this study aims to explore the current knowledge and attitude status as well as the determinants associated with viral infections in our setting. This might bring substantial changes to the ongoing threat of disease and the critical need for well‐informed healthcare workers who can effectively mitigate the spread of the virus in high‐risk environments.

## 2 Methods and Materials

### 2.1 Study Design, Period, Area, and Setting

A cross‐sectional study design was employed between June 13th and September 3rd, 2024 to assess the knowledge, attitudes, and associated factors of healthcare professionals regarding Mpox at Debre Tabor Comprehensive Specialized Hospital (DTCSH), located in Debre Tabor (https://en.wikipedia.org/wiki/Debre_Tabor), Northwest Ethiopia. DTCSH is located in the capital city of the South Gondar Zone, Amhara region of Ethiopia. It is a major tertiary health facility that provides comprehensive medical services and specialized care to a population of 2,484,929, which includes 1,257,323 men and 1,227,606 women [27]. The hospital is renowned for its dedication to enhancing health outcomes by integrating clinical excellence, community involvement, and research initiatives. It plays a crucial role in tackling various health challenges faced by the community, especially in the areas of maternal and child health, infectious diseases, and chronic conditions. The institution collaborates with Debre Tabor University to ensure high‐quality care is delivered by skilled healthcare professionals through integrated research initiatives and ongoing professional development.

The South Gondar zone is home to 405 health posts, 96 health facilities, 8 basic hospitals, and 1 comprehensive specialized hospital. This zone encompasses 4 town administrations and 14 districts. DTCSH employs over 600 healthcare professionals across various departments. The facility provides more than 300 beds in different wards, including pediatric, medical, surgical, gynecological, obstetric, neonatal, intensive care, emergency, ophthalmic, and psychiatric units. Debre Tabor is situated at a latitude of 11.850° N and a longitude of 38.017° E, with an elevation of 2706 meters (8878 feet) above sea level.

### 2.2 Study Population

The study population included all healthcare professionals working at DTCSH during the study period, including physicians, nurses, laboratory technicians, pharmacists, and other allied healthcare workers.

### 2.3 Inclusion and Exclusion Criteria

The inclusion criteria for this study were healthcare professionals who had been working at DTCSH for at least 6 months. It includes those who were working in various departments (inpatients and outpatients) including medical, surgical, emergency, intensive care unit, pediatric, labor, and laboratory facilities. Healthcare professionals who were available during the study period and willing to participate in the study were also involved. On the contrary, healthcare providers who were on leave during the data collection period and refused to participate were excluded. Additionally, nonclinical staff who were not directly involved in patient care, as well as individuals without formal education or training in healthcare, were excluded.

### 2.4 Sample Size Determination and Sampling Technique

The sample size for this study was calculated using a single population proportion formula:

$$n=\frac{Z_{\alpha /2}^{2}\cdot p\cdot (1-p)}{d^{2}},$$

where *n* = total sample size.

*z* = the standard variation at 95% confidence interval and, *α* = 1.96, and *d* ^2^ = margin of error (*d* = 0.05).

*p* = Assuming the proportion of healthcare professionals with good knowledge about Mpox was 50% (no prior studies in our setting) then, *n* = (1.96)^2^ × 0.5 (1−0.5)/(0.05)^2^, *n* ≈ 384. Convenience sampling was used to choose participants for this study. Based on the aim of the study, a variety of healthcare professionals, including clinicians, nurses, laboratory technologists, pharmacists, midwives, and other healthcare professionals working at different departments were conveniently selected.

### 2.5 Data Collection Tools and Procedure

**Figure 1:**
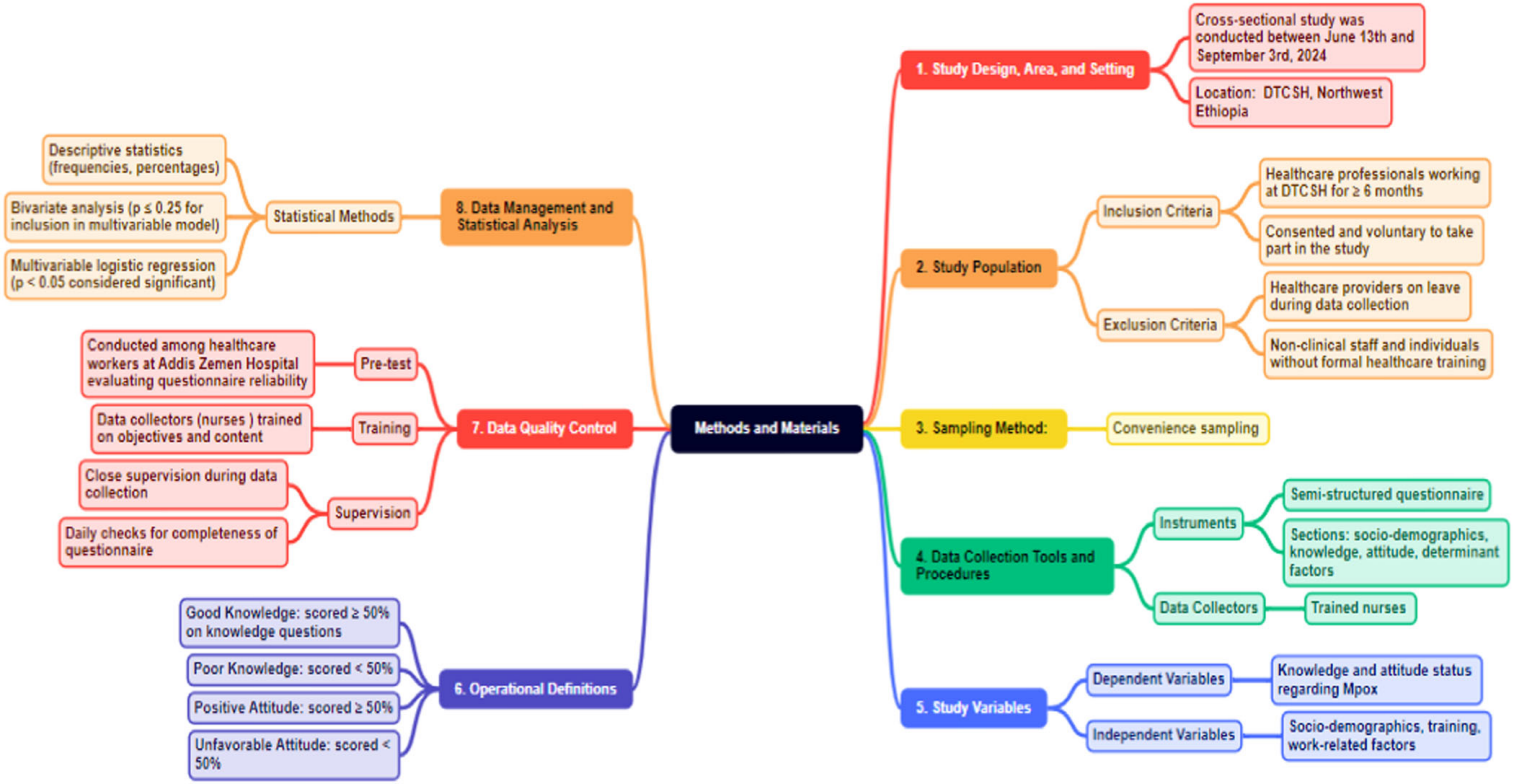
A comprehensive and schematic illustration of the study's method and material.

Before data collection, a brief study information sheet and written consent form were provided to the volunteer study participants. Then, data were collected using semi‐structured questionnaire. The questionnaire was developed by the team of authors after a thorough relevant literature review. The scope of the questionnaire includes socio‐demographic characteristics (e.g., age, sex, profession, years of experience, level of education), knowledge assessment (transmission, clinical presentation, laboratory diagnosis, choice of specimen, prevention, and control) of Mpox. Besides, attitude assessments such as healthcare professionals' perceived susceptibility, seriousness, vaccine acceptance, and willingness to engage in prevention and control practices were included. Furthermore, questions concerning determinant factors, such as exposure to Mpox patients, use of personal protective equipment, and participation in research or tailored infection control training were developed. The comprehensive and schematic illustration of the study's methods and materials is given in (Figure 1).

### 2.6 Study Variables

The knowledge and attitude status of participants regarding Mpox were categorized as dependent variables. On the other hand, independent study variables include socio‐demographic characteristics, relevant professional training, and work‐related factors.

### 2.7 Operational Definitions

#### 2.7.1 Good Knowledge

Participants who scored 50% or higher on all knowledge questions. In closed‐ended questionnaires, each correct response was assigned a score of 1, while an incorrect or uncertain response was scored as 0. In open‐ended or multiple‐choice questionnaires, participants who provided at least one appropriate answer were scored one to considered knowledgeable.

#### 2.7.2 Poor Knowledge

A 0%–49% score was considered poor/unfavorable knowledge.

#### 2.7.3 Favorable Attitude

A mean score of ≥ 50% was assigned a favorable attitude while below was an unfavorable attitude.

### 2.8 Data Quality Control

For this study, the questionnaire was initially developed in the English language and subsequently translated into the local language (Amharic version). To enhance its clarity, simplicity, completeness, consistency, and accuracy, it was then back transcribed into English version. Besides, the questionnaire was pre‐tested on healthcare professionals working outside of the study facility (Addis Zemen Hospital in South Gondar) to ensure that it was clear, complete, and simple prior to the actual data collection. Modifications were then made based on the feedback received. Moreover, data collectors (nurses) were trained by the team of authors regarding the study's objectives and the questionnaire's content. A panel of investigators closely supervised them during the data collection process, and the principal investigator conducted daily checks for the completed questionnaires.

### 2.9 Data Management and Statistical Analysis

The data was entered into SPSS version 25 for statistical analysis. Descriptive statistics, such as frequencies, percentages, confidence intervals, and standard deviations, were meticulously calculated. Data for socio‐demographic characteristics, knowledge, attitudes, and associated factors were thoroughly summarized using text, tables, and figures. The relationships between the explanatory and dependent variables were rigorously assessed using a two‐step logistic regression model. Firstly, bivariate analysis was performed on all variables to evaluate the relationships between the outcome variables (knowledge and attitude levels) and explanatory variables. Then, only variables with a *p* value of ≤ 0.25 in the bivariate analysis were included in the multivariable logistic regression model to identify independent predictors of knowledge and attitude status. A p‐value below 0.05 was considered to indicate a statistically significant association.

## 3 Ethical Clearance

Ethical approval for this study was granted by the research and community service central committee of the College of Health Sciences (Reference Number: CHS3419/2024), Debre Tabor University. The research was conducted following the Declaration of Helsinki, which outlines ethical principles for medical research involving human subjects. Permission to carry out the study was also obtained from the administration of DTCSH. Before data collection informed written consent was secured from each participant. To ensure confidentiality, data collection was conducted using study‐coded identifiers, and all collected data were stored securely, with access limited to authorized research personnel.

## 4 Results

A total of 384 healthcare professionals participated in this study. The overall response rate of this study was 100%. The majority of the participants were male (55.47%), and the largest age group was 31–39 years, making up 33.85% of the participants, followed by the 26–30 years' group (21.88%). The mean age of the study participants was 33 ± 7.98 (range: 20–50 years). In terms of healthcare roles, nurses were the largest group (Table 1), comprising 31.25% of the participants, followed by medical doctors (22.92%) and midwives (17.45%). The professionals were assigned to different wards, with the medical ward being the most common (21.09%), followed by surgical (16.67%) and pediatric (14.58%) wards. Concerning work experience, 32.55% had 11–15 years of experience, while 30.47% had 1–5 years.

**Table 1:** Sociodemographic characteristics of healthcare works.

R. No	Study variables	Category	Frequency (*N* = 384)	Percentage (100%)
1	Gender	Male	213	55.47
		Female	171	44.53
2	Age (years)	20–25	78	20.31
		26–30	84	21.88
		31–39	130	33.85
		> = 40	92	23.96
3	Marital status	Single	119	30.99
		Married	210	54.69
		Divorced	55	14.32
4	Level of education	Bachelor degree	150	39.06
		Master's degree	89	23.18
		Doctorate (PhD)	67	17.45
		Diploma	78	20.31
5	Health profession	Medical Laboratory	33	8.59
		Medical Doctor	88	22.92
		Anesthetist	19	4.95
		Nurse	120	31.25
		Pharmacy	22	5.73
		Midwifery	67	17.45
		Others^#^	35	9.11
6	Professional engagement	Inpatient departments	71	18.49
		Outpatient departments	100	26.04
		Both department	213	55.48
7	Assigned wards	Pediatric	56	14.58
	( ≥ 6 months)	Intensive care unit	47	12.24
		Medical	81	21.09
		Surgical	64	16.67
		Gynecology	53	13.80
		Others*	88	22.92
8	Work experience (years)	1–5	117	30.47
		6–10	98	25.52
		11–15	125	32.55
		> = 16	44	11.46

*Note:* Others^#^, environmental science, psychiatry, radiology; Others*, dental, ophthalmic, orthopedic.

### 4.1 Knowledge and Attitude Assessment Towards Mpox

**Figure 2:**
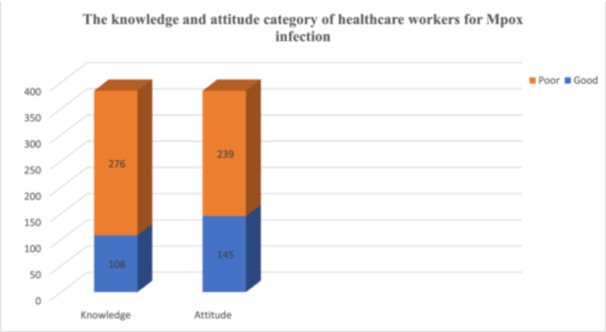
The knowledge and attitude level of healthcare professionals regarding Mpox at DTCSH, Northwest Ethiopia, 2024.

In this study, a total of 46 item questions were used to assess healthcare professionals' comprehension and perceptions concerning Mpox. The questions include 12 (general), 07 (transmission), 04 (clinical presentation), 02 (source of the specimen), 04 (diagnosis) and 17 (treatments and preventions). The study revealed that only 108 (28.13%) of healthcare professionals were knowledgeable about Mpox. Additionally, 145 (37.76%) of participants had a favorable attitude towards Mpox (Figure 2). Around 75.26% correctly identified it as a viral infection. The study also found that 92.97% of participants recognized Mpox as a contagious disease (Table 2). Regarding attitudes towards Mpox, a majority (59.64%) did not recognize Mpox as a PHEIC. In this study, 170 (44.27%), and 145 (37.76%) of the study participants considered swabs and PCR as the preferred clinical specimen and laboratory test for diagnosis, respectively. Risk perception varied among respondents. Only 24.48% identified males as being at greater risk. Additionally, 52.34% indicated that they would refuse the Mpox vaccine for themselves, and 54.17% were unlikely to encourage others to get vaccinated.

**Table 2:** Assessment of knowledge, and attitude of healthcare professionals regarding Mpox using categorically designed questions with their alternative responses.

Item category	Specific questions	Alternatives	Frequency (*N* = 384)	Percentage (100%)
**General**	Have you heard about Mpox infections	Yes	230	59.90
		No	154	40.10
	What type is Mpox infection	Viral	289	75.26
		Bacterial	95	24.74
		Fungal	0	0.00
		Parasite	0	0.00
	Is Mpox contagious	Yes	357	92.97
		No	27	7.03
	How many common clades exist in the Mpox virus	One	89	23.18
		Two	175	45.57
		Three	120	31.25
	Have you taken any training on Mpox infection	Yes	0	0.00
		No	384	100.00
	Is Mpox PHEIC (pandemic)	Yes	155	40.36
		No	229	59.64
	Have you encountered Mpox cases in your professional experience	Yes	0	0.00
		No	384	100.00
	Who is at risk for Mpox	Children	146	38.02
		Adults	28	7.29
		Elderly people	98	25.52
		All	112	29.17
	Is gender disparity attributed to the risk of infection	Yes	110	28.65
		No	200	52.08
		Do not know	74	19.27
	Based on gender, who is at risk for Mpox	Male	94	24.48
		Female	300	78.13
		Both	10	2.60
	Are people with multiple sexual partners at higher risk of infections	Yes	241	62.76
		No	143	37.24
	Can Mpox cause an extra burden on the healthcare system in Ethiopia?	Yes	317	82.55
		No	67	17.45
**Transmission**	Which transmission vehicles are common	Human contact	78	20.31
		Animal contact	100	26.04
		Bite of insects	18	4.69
		Air born	117	30.47
		Food & water born	49	12.76
		Others^&^	22	5.73
	Are contaminated medical equipment and inanimate surfaces possible sources of infection	Yes	129	33.59
		No	255	66.41
	Is Mpox able to survive for several days on contaminated surfaces & equipment	Yes	86	22.40
		No	298	77.60
	Can rash physical contact be transmissible	Yes	320	83.33
		No	64	16.67
	Can Mpox spread during pregnancy to the fetus	Yes	111	28.91
		No	273	71.09
	When does it transmit	During birth	281	73.18
		After birth	100	26.04
		I do not know	3	0.78
	Is Mpox zoonotic disease	Yes	319	83.07
		No	65	16.93
**Clinical presentation**	Are Mpox and smallpox clinically different	Yes	370	96.35
		No	14	3.65
	Can you distinguish Mpox from chickenpox, measles, bacterial skin infections, scabies, and herpes	Yes	299	77.86
		No	85	22.14
	What are the possible signs and symptoms	Skin rash	188	48.96
		Sore throat, headache, muscle aches	84	21.88
		Swollen lymph nodes	33	8.59
		Fever	79	20.57
	Do you think Mpox will cause a more severe disease than smallpox	Yes	200	52.08
		No	184	47.92
**Source of specimen**	What is the preferred source of specimen	Urine and stool	42	10.94
		Skin lesions	71	18.49
		Body fluids	36	9.38
		Blood	90	23.44
		Swabs	145	37.76
	When is the preferred time for specimen collection during Mpox outbreak?	Before symptom onset	40	10.42
		At the onset of symptoms	155	40.36
		During the peak of symptoms	112	29.17
		After symptoms have subsided	77	20.05
**Diagnosis**	What is the preferred lab test for Mpox	Microscopy	69	17.97
		GeneXpert	22	5.73
		PCR	170	44.27
		Viral culture	88	22.92
		Others*	35	9.11
	Can antibody detection methods distinguish between different Orthopoxviruses	Yes	199	51.82
		No	185	48.18
	How important is surveillance during an outbreak	Very important	285	74.22
		Somewhat important	99	25.78
		Not important at all	0	0.00
	Can the genomic sequencing distinguish Mpox variants	Yes	318	82.81
		No	66	17.19
**Treatments and preventions**	Can wearing masks, and using sanitizer prevent Mpox transmission	Yes	216	56.25
		No	100	26.04
		I do not know	68	17.71
	Is hand hygiene essential to contain Mpox infections	Yes	300	78.13
		No	84	21.88
	Is environmental sanitation compulsory to mitigate Mpox infections	Yes	173	45.05
		No	211	54.95
	Are PPE measures effective in preventing Mpox	Yes	275	71.61
		No	109	28.39
	Is Mpox self‐limited disease	Yes	92	23.96
		No	200	52.08
		I do not know	92	23.96
	Is paracetamol one management	Yes	82	21.35
		No	302	78.65
	Are there effective antiviral medications	Yes	290	75.52
		No	90	23.44
		Not sure	4	1.04
	Is there a vaccine available for Mpox?	Yes	119	30.99
		No	176	45.83
		I do not know	89	23.18
	Do you think Mpox vaccine is safe	Yes	214	55.73
		No	170	44.27
	Is the smallpox vaccine protective against Mpox	Yes	110	28.65
		No	274	71.35
	Is there a specific vaccine for Mpox?	Yes	119	30.99
		No	265	69.01
	Will you accept Mpox vaccine	Yes	183	47.66
		No	201	52.34
	Will you encourage others to accept vaccination	Yes	176	45.83
		No	208	54.17
	Have you been vaccinated against Mpox or smallpox viruses over the last 5 years	Yes	0	0.00
		No	384	100.00
	Can vaccination duration vary based on symptoms	Yes	116	30.21
		No	268	69.79
	Who is more eligible to get the vaccination during outbreak	Health workers at risk of exposure	180	46.87
		People with multiple sex partners	87	22.66
		Sex workers.	110	28.65
		Others^%^	7	1.82
	What is your plan during an outbreak of Mpox	Patient isolation	164	42.71
		Adherence to safety precautions	98	25.52
		Case notification	122	31.77

Abbreviations: Mpox, Monkeypox virus; Others^%^, elderly people, children, pregnant women; Others^&^, Contaminated materials/surfaces, contaminated clothes); Others*, ELISA, imaging, and clinical diagnosis; PCR; polymerase chain reaction; PHEIC; public health emergency of international concern; PPE, personal protective equipment.

### 4.2 Associated Factors Analysis

The multivariate logistic regression analysis (Table 3) indicates that age groups (26–30 years) and prior knowledge of Mpox are the most significant predictors of good knowledge and favorable attitudes toward the infection among healthcare professionals. Healthcare workers aged 26–30 years were significantly more likely to have good knowledge (AOR = 2.99, *p* < 0.001) and maintain a favorable attitude towards Mpox. Prior awareness of Mpox also strongly influenced both knowledge and attitude, with those already familiar with the virus being more likely to have favorable comprehension and perception (AOR = 1.45, *p* = 0.002).

**Table 3:** Bivariate and multivariate logistic regression analysis of the factors associated with the knowledge of healthcare professionals toward Mpox.

Explanatory variables	Groups	Mpox knowledge status		Binary logistic regression	Multiple logistic regression	
		Good no (%)	Poor no (%)	COR (95% CI)	AOR (95% CI)	*p* value
Gender	Male	32 (15.02)	181 (84.98)	1	1	
	Female	39 (22.80)	132 (77.20)	1.45 (0.75–2.18)	0.67 (0.32–3.06)	0.06
Age	20–25	28 (35.90)	19 (24.36)	1	1	
	26–30	16 (19.05)	68 (80.95)	1.33 (0.99–4.82)	2.99 (0.45–4.66)	**< 0.001**
	31–39	15 (11.54)	115 (88.46)	1.02 (1.00–1.88)	1.44 (0.28–1.77)	0.54
	≥ 40	9 (9.78)	83 (90.22)	0.34 (0.11–1.23)	0.41 (0.33–1.89)	0.26
Work experience (year)	1–5	55 (47.00)	62 (53.00)	1	1	
	6–10	45 (45.92)	44 (54.08)	1.83 (0.39–2.99)	0.39 (0.45–1.76)	0.82
	11–15	50 (40.00)	75 (60.00)	0.62 (0.05–1.58)	1.94 (0.78–3.17)	0.66
	≥ 16	19 (43.18)	25 (56.82)	1.04 (0.12–1.80)	0.71 (0.33–3.19)	0.49
Have you heard about Mpox	Yes	89 (38.70)	141 (61.30)	1.09 (0.04–2.09)	1.45 (0.87–2.65)	**0.002**
	No	66 (42.86)	88 (57.14)	1	1	

*Note: p* < 0.05 (bolded) indicate a statistically significant association.

Abbreviations: AOR, adjusted odds ratio; CI, confidence interval; COR, crude odds ratio.

Meanwhile, an attitude factorial analysis of healthcare professionals has revealed that participants in the age group of 26–30 demonstrate remarkably associated favorable attitudes [AOR & 95% CI (0.40 (0.25–0.82), *p* = 0.03)] towards Mpox (Table 4).

**Table 4:** Bivariate and multivariate logistic regression analysis of the factors associated with the attitude of healthcare professionals toward Mpox.

Explanatory variables	Groups	Mpox attitude status		Binary logistic regression	Multiple logistic regression	
		Favorable no (%)	Unfavorable no (%)	COR (95% CI)	AOR (95% CI)	*p* value
Gender	Male	74 (43.27)	97 (56.73)	1	1	
	Female	87 (50.88)	84 (49.12)	0.216 (0.11–1.42)	0.65 (0.40–1.02)	0.08
Age	20–25	59 (75.64)	19 (24.36)	1	1	
	26–30	25 (29.76)	59 (70.24)	0.41 (0.34–1.28)	0.40 (0.25–0.82)	**0.03**
	31–39	66 (50.77)	64 (49.23)	3.12 (0.94–4.37)	2.25 (2.10–3.10)	0.16
	≥ 40	55 (59.78)	37 (40.22)	1.41 (1.01–2.06)	1.15 (1.02–3.55)	0.97
Work experience (year)	1–5	35 (29.91)	82 (70.09)	1	1	
	6–10	39 (39.80)	59 (60.20)	0.57 (0.22–2.10)	0.55 (0.35–2.10)	0.75
	11–15	67 (53.60)	58 (46.40)	0.70 (0.45–0.87)	0.60 (0.25–0.90)	0.28
	≥ 16	24 (54.54)	20 (45.45)	1.03 (0.79–1.81)	1.10 (0.75–2.45)	0.71
Have you heard about Mpox	Yes	85 (36.96)	145 (63.04)	0.21 (0.11–10.45)	0.82 (0.67–2.30)	0.95
	No	70 (45.45)	84 (54.54)	1	1	

*Note: p* < 0.05 (bolded) indicate a statistically significant association.

Abbreviations: AOR, adjusted odds ratio; CI, confidence interval; COR, crude odds ratio.

## 5 Discussion

Mpox, a zoonotic viral disease has become a global health emergency. The disease has historically been confined to parts of West and Central Africa [28]. However, the current outbreak has seen the virus spread to many nonendemic countries, leading the WHO to declare a PHEIC [12]. The recent global outbreak has emphasized the significance of understanding the knowledge and attitudes of healthcare professionals toward this infectious disease pandemic. Healthcare workers are essential in detecting, diagnosing, and managing cases of the disease, as well as implementing necessary public health measures to control the virus's spread [29]. Many healthcare providers have been found to lack the knowledge and experience needed to effectively manage Mpox cases. This is especially worrying due to the potential for significant illness and death from Mpox infections, as well as the limited availability of specific treatments and vaccines [28, 30].

In the current study, 28.13% of healthcare workers (HCWs) demonstrated good knowledge, while 37.76% exhibited favorable attitudes towards Mpox. A study conducted in Cameroon reported that 42.1% of HCWs showed good knowledge about Mpox. This percentage is notably higher than that found in the present study, suggesting that HCWs in Cameroon may have had greater exposure or better access to information about Mpox. The higher percentage indicates the potential impact of regional differences in educational initiatives and public health communication. Another study conducted in Southern Italy showed [23] that less than two‐thirds of HCWs were able to accurately define Mpox, and only 22.8% recognized contact with contaminated objects as a transmission route. The overall average knowledge score was 3.4 out of 9, indicating a consistent trend of insufficient knowledge as observed in the current study. The Italian study reveals specific gaps in understanding transmission routes, which is consistent with the lower percentage of HCWs with good knowledge in the present study. In a combined analysis of 27 studies in Arabic regions [24], the study found that 33% of HCWs had good knowledge, and 40% had a positive attitude. These numbers are similar to the 28.13% and 37.76% reported in our study, showing consistent trends in knowledge and attitudes across different regions. The slight variance may be due to differences in healthcare infrastructure, training opportunities, and access to information [31, 32].

In a study conducted in Bangladesh, 45.5% of medical doctors demonstrated good knowledge about Mpox, and 52.2% showed a positive attitude. These percentages are higher than those found in the current study, indicating that factors such as higher education levels and previous training, which were significant in Bangladesh, might contribute to better outcomes in that region [29]. In Nigeria, 60.5% of respondents exhibited adequate knowledge of Mpox, yet only 28.9% held a positive perception of it. Although the level of knowledge is notably higher than what is observed in this study, the proportion of individuals with a positive perception is lower than the favorable attitudes identified here. This discrepancy underscores the intricate relationship between knowledge and attitude, demonstrating that possessing good knowledge does not necessarily lead to a positive attitude [33].

In a study conducted in Ethiopia [21], 38.5% of HCWs demonstrated good knowledge about Mpox, while 62% displayed positive attitudes. In comparison to the present study, the proportion of HCWs with good knowledge in Ethiopia is slightly higher (38.5% vs. 28.13%), and the favorable attitude is significantly greater (62% vs. 37.76%). This indicates that Ethiopian HCWs may have benefited from more effective training or better access to Mpox‐related information, which could enhance their preparedness and attitudes. Conversely, in a study from Saudi Arabia, HCWs recorded an average knowledge score of 9.90, deemed poor, with widespread misconceptions about transmission routes. Despite this lack of knowledge, they achieved an average attitude score of 50.19, indicating an overall positive outlook towards Mpox prevention. This knowledge gap in Saudi Arabia reflects the findings in the present study, where only a small percentage of HCWs possess good knowledge. However, the more favorable attitudes observed in Saudi Arabia (compared to 37.76% in the current study) suggest that attitudes may not be entirely influenced by levels of knowledge [34]. A similar study conducted among HCWs in Nepal revealed that 60.4% had a high level of knowledge about Mpox, while 51.1% demonstrated a positive attitude towards it. The knowledge percentage in Nepal is more than double that observed in the present study, emphasizing a considerable gap. Additionally, the percentage of HCWs with a positive attitude is higher, suggesting that educational and awareness initiatives in Nepal may be more effective, or that HCWs there may be more receptive to public health messages [35]. In Lebanon [36], 33.7% of HCWs demonstrated knowledge of Mpox at or above the 75%, revealing significant gaps in understanding. This percentage is comparable to the findings of the present study, where 28.13% exhibited good knowledge, indicating similar challenges in effectively disseminating information. However, the presence of conspiracy beliefs in Lebanon, which adversely affected attitudes, suggests that cultural and social factors may influence HCWs' perceptions more in that context than in the current study. Interestingly, the global meta‐analysis [37] found that 26.0% of HCWs possessed good knowledge while 34.6% held positive attitudes towards Mpox. These figures closely align with the present study's findings of 28.13% good knowledge and 37.76% favorable attitudes. This consistency indicates that the issues identified in the current study are reflective of a larger global trend, wherein HCWs across different regions face similar levels of misinformation and unpreparedness regarding Mpox. This underscores the necessity for comprehensive global strategies to enhance education and training efforts.

Furthermore, in a study conducted among clinicians in the USA [38], findings revealed relatively low levels of knowledge about Mpox, with only about 25% of participants having prior awareness of the disease. The attitudes towards Mpox control were mixed, and there was a lack of sufficient intention to adopt preventive measures. These results are closely aligned with the present study's findings of 28.13% exhibiting good knowledge, indicating that even in high‐resource settings like the USA, there are considerable gaps in Mpox knowledge and preparedness among healthcare professionals. In China, 42% of male sex workers demonstrated good knowledge about Mpox, and 58% expressed a willingness to receive the Mpox vaccine. Although this demographic differs from HCWs, their knowledge level surpasses the 28.13% recorded in the present study. Additionally, the higher willingness to vaccinate among this group contrasts with the less favorable attitudes observed in HCWs in the current study (37.76%). This suggests that targeted educational initiatives can enhance both knowledge and attitudes, even among nonhealthcare worker populations [39]. These differences highlight the need for enhanced educational interventions and targeted training programs for HCWs to improve their knowledge and attitudes, thereby better preparing them for managing Mpox and similar emerging diseases. The data suggest that regions with better outcomes may have more robust public health infrastructures or more effective training and education programs in place [40, 41].

In the present study, participants in the age group of 26–30 years and those with prior knowledge of Mpox were significantly associated with both good knowledge and favorable attitudes. Specifically, HCWs aged 26–30 were significantly more likely to possess good knowledge (AOR = 2.99, *p* < 0.001) and maintain a positive attitude. Furthermore, prior awareness of Mpox was strongly linked to both knowledge and attitude, with those already familiar with the virus being more likely to have a favorable understanding and perception (AOR = 1.45, *p* = 0.002). A study in Bangladesh [29], found that higher education levels (AOR = 2.1, 95% CI: 1.5–2.9) and previous training on emerging infectious diseases (AOR = 1.8, 95% CI: 1.3–2.5) were significantly associated with good knowledge of Mpox. Similarly, professional experience and working in urban areas were linked to positive attitudes. This comparison suggests that both the present study and the Bangladeshi study recognize the importance of prior knowledge and experience in shaping HCWs' understanding and attitudes. However, while the Bangladesh study highlighted the role of education and urban work environments, the present study placed greater emphasis on age as a determining factor. The Lebanon study suggests [36] that while demographic factors like age are important, other factors like education, experience, and social beliefs can significantly influence knowledge and attitudes. Another study in the Arabic region [24] has shown that factors such as age, professional experience, and level of education were significant determinants of knowledge. Older HCWs and those with more professional experience tended to have better knowledge. A similar study in Nigeria [33] indicated significant factors associated with knowledge levels included age (*p* = 0.020), educational qualification (*p* = 0.004), and occupation. Importantly, another study in Ethiopia [25] that having a master's degree or above was significantly associated with good knowledge (AOR = 11.25; 95% CI: 2.03–62.33). in the study, similarly, access to information about Mpox was another key determinant (AOR = 3.37; 95% CI: 1.33–8.50). furthermore, a study in Nepal [42] revealed higher education levels were significantly associated with better knowledge (AOR = 3.12; 95% CI: 1.75–5.56). More experience in healthcare was also associated with higher knowledge levels (AOR = 2.45; 95% CI: 1.30–4.60). Participants who had received training on infectious diseases had significantly better knowledge (AOR = 4.20; 95% CI: 2.10–8.40). In Cameron [26], younger individuals aged 26‐30 were associated with good knowledge, while workplace type correlated with excellent knowledge of Mpox (AOR [95% CI]: 4.01 [1.43–11.24]).

## 6 Strengths and Limitations

This study explores the knowledge, attitude, and factors associated with Mpox among healthcare professionals in our setting. It comprehensively provided a better insight into a very timely public health concern and how prepared and concerned the frontline healthcare workers were in a resource‐constrained setting. This is because increased clinical skills and professional capabilities are essential to contain infectious diseases promptly. The focus on a specialized hospital will enhance its relevance, as it is clear that the role of specialized institutions is crucial in the management of emerging infectious diseases. Moreover, the comprehensive approach used in this study will provide a baseline perspective for future research on Mpox in Ethiopia. However, this study has some limitations. Being single‐center research, the findings may not generally represent other regions or healthcare facilities that have different sociodemographics. The reliance on self‐reported data might introduce tendencies of response bias and, therefore, could affect the accuracy of the reported knowledge and attitudes scores. Additionally, cross‐sectional study design limits the ability to infer the identified factors and outcomes in terms of a causal relationship. Despite such limitations, the study will provide important groundwork that may contribute to future studies and interventions into Mpox preparedness and management in similar settings.

## 7 Conclusions and Recommendations

The study reveals significant knowledge and attitude gaps among HCPs regarding Mpox, with only 28.13% exhibiting a strong understanding of the disease. While 37.76% of HCPs showed positive attitudes towards Mpox, a large number were inadequately equipped to participate in preventive and control measures. This shortfall is particularly alarming in light of the global increase in Mpox cases and its designation as a PHEIC by the WHO. Moreover, the study emphasized a specific lack of training on Mpox among participants, as none reported any prior training on the topic, pointing to a grave lack of readiness for pandemic response. Further analysis revealed that sociodemographic factors, especially age and previous awareness, were significantly associated with knowledge and attitudes levels; younger HCPs (aged 26–30) and those who had prior Mpox‐related information demonstrated greater knowledge and a more favorable attitude.

To address these gaps, the study alarms for the context‐specific establishment of educational programs focused on Mpox and other emerging infectious diseases of public health priority. These initiatives should be specifically designed to fill identified knowledge gaps and primarily target younger healthcare providers as well as those lacking relevant training. It is also essential to prioritize ongoing professional development through regular workshops, seminars, and online courses to ensure all HCPs remain informed about the latest updates on Mpox, which should include practical training on disease identification, diagnosis, and management strategies. Moreover, campaigns aimed at shifting attitudes are necessary to enhance HCPs' perspectives on Mpox, highlighting the significance of vaccination, disease prevention, and the implications for global health. Further research is advised to delve into the root causes of these knowledge and attitude deficiencies, which could pave the way for more effective interventions. The formulation and execution of clear policy guidelines for Mpox management within healthcare facilities are also vital, encompassing protocols for early detection, isolation, treatment, and safeguarding healthcare workers. Lastly, fostering interdisciplinary collaboration among various HCPs can strengthen the overall response to Mpox outbreaks, encouraging knowledge exchange and a more coordinated patient care strategy.

## Author Contributions

**Teklehaimanot Kiros:** conceived and designed the experiments; analyzed and interpreted the data; wrote the paper. **Mulat Erkihun, Bekele Sharew, Andargachew Almaw, Ayenew Assefa, Ayenew Berhan, Tegenaw Tiruneh, Birhanemaskal Malkamu, Eninur Dejen, Mahider Shimelis, Alemie Fentie, Mitikie Wondmagegn, Dessie Tegegne, Birhanu Getie, Yenealem Solomon, and Shewaneh Damtie:** analyzed and interpreted the data; wrote the paper. All authors have read and approved the final version of the manuscript and the corresponding author (Teklehaimanot Kiros) had full access to all of the data in this study and takes complete responsibility for the integrity of the data and the accuracy of the data analysis.

## Ethics Statement

The authors have nothing to report.

## Conflicts of Interest

The authors declare no conflicts of interest.

## Transparency Statement

The lead author Teklehaimanot Kiros affirms that this manuscript is an honest, accurate, and transparent account of the study being reported; that no important aspects of the study have been omitted; and that any discrepancies from the study as planned (and, if relevant, registered) have been explained.

## Data Availability

We confirm that all data underlying the results presented are available in the article. The authors confirm that the data supporting the findings of this study are available within the article [and/or] its supplementary materials.
